# Design and Optimization of SAR Signal Array Receiving Based on MOEA/D-HPSO

**DOI:** 10.3390/s26123879

**Published:** 2026-06-18

**Authors:** Zhiyang Zhang, Hongji Xing, Ximing Yu, Xiaogang Tang

**Affiliations:** School of Space Information, Space Engineering University, Beijing 101416, China

**Keywords:** spaceborne SAR, wide-angle scanning, non-uniform planar array, multi-objective optimization, Pareto front, array pattern synthesis

## Abstract

Passive reception of spaceborne synthetic aperture radar (SAR) signals is of great significance for acquiring target characteristics and identifying SAR operating states. With the rapidly growing demand for high-quality SAR signal reception, signal-receiving arrays are prone to beam performance deterioration and difficulty in beamforming under wide-angle scanning conditions. Traditional uniform arrays fail to meet practical engineering requirements and cannot balance multiple conflicting performance indicators. To address the above technical bottlenecks, this paper proposes a design method of a non-uniform planar receiving array based on the MOEA/D-HPSO algorithm. Taking maximum sidelobe level (MSL), array gain (G), and beamwidth (BW) as core performance indicators, a multi-objective optimization model of SAR signal-receiving array for wide-angle scanning is established. This method integrates the multi-objective decomposition strategy and hybrid genetic particle swarm optimization mechanism, decomposes complex multi-objective problems into several scalar subproblems, obtains uniformly distributed Pareto fronts, and effectively improves the diversity of solution sets. Simulation experimental results show that the proposed algorithm is superior to traditional mainstream algorithms such as NSGA-II and MOEA/D-DE in terms of convergence accuracy, solution set distribution, and various performance indicators. Typical array design examples verify that the proposed method can adapt to various engineering application scenarios and provide technical support for spaceborne SAR signal reception and spectrum management.

## 1. Introduction

SAR is a core technology for space-based Earth observation [1], usually deployed on satellites and other spacecraft. Effective signal receiving is essential for high-precision positioning, identification, and spectrum management. Since most spaceborne SAR operates in low Earth orbit (LEO), its angle relative to the receiving system varies widely during transit, causing serious beam degradation and poor adaptability in the receiving system. This increases receiving difficulty and raises the demand on receiver design. Conventional single-antenna receivers only obtain time–frequency information without spatial dimension [2], making multi-channel coherent processing and accurate signal sorting difficult.

Array antennas can simultaneously collect multi-channel data and measure phase differences, supporting DOA estimation, signal sorting, and coherent processing. Thus, they are widely used in high-precision receiving systems. However, traditional uniform full arrays lack beam control flexibility and suffer from severe grating lobes, which mask useful signals. Therefore, designing non-uniform planar arrays for flexible wide-angle beamforming is essential.

These issues fall into the multi-objective optimization framework. The key challenge is selecting appropriate array pattern indicators for effective beamforming and using efficient intelligent optimization algorithms to arrange elements, breaking periodic geometric structures [3] to achieve satisfactory receiving performance. Reference [4] uses a matrix constraint method to realize the element constraint conditions for practical sparse planar arrays. Reference [5] combines machine learning and an improved genetic algorithm to minimize the MSL. Reference [6] optimizes array synthesis via matrix mapping to reduce sidelobes. Reference [7] uses particle swarm optimization to generate desired sector beams and improve beam quality. Reference [8] optimizes element positions through sequential convex programming to balance angular resolution and energy utilization. For non-uniform arrays, angular ambiguity function and expanded beam pattern analyses offer more accurate resolution assessment [9]. Moreover, the BW can be directly optimized under given sidelobe constraints using alternating-direction multiplier methods, achieving better angular resolution than uniform arrays with the same aperture [10]. Reference [11] optimizes subarray-based large-aperture antenna systems by trading off BW and MSL, yielding better DOA estimation performance. Beyond array geometry optimization, joint beam and time–frequency processing has also enabled instantaneous positioning with a single satellite [12]. Existing numerical optimization methods mainly improve or hybridize traditional algorithms. Examples include improved genetic algorithms with differential crossover [13], optimized sparse linear arrays using modified particle swarm optimization [14], and hybrid algorithms balancing exploration and exploitation [15,16]. These methods perform well in array optimization. Nevertheless, for multiple conflicting indicators, a single optimal solution cannot satisfy specific engineering preferences. Traditional methods lack flexible trade-off strategies and weight configuration, making them unable to meet fast and targeted receiving requirements under wide satellite scanning angles.

This paper presents an array design method for wide-angle scanning, which adapts to various receiving requirements by jointly optimizing G, MSL, and BW. A hybrid particle swarm optimization is utilized for multi-objective optimization to generate multiple Pareto solutions for engineering decision-making. The proposed method achieves superior beam pattern performance indicators while rapidly adapting to different scenarios, effectively extending the receiving time window and laying a solid foundation for subsequent processing.

The main contributions of this paper are as follows:

1. Traditional MOEA/D uses only a single aggregation function; the proposed method sequentially adopts a two-stage aggregation strategy (weighted sum followed by Tchebycheff) to handle the non-convex Pareto front, balancing global exploration and precise convergence.
2. Standard PSO relies on a single population for global search, whereas this paper embeds HPSO into the MOEA/D framework, equipping each subproblem with an independent PSO swarm, thereby enhancing targeted search for each subproblem.
3. Conventional decomposition-based algorithms (e.g., MOEA/D and MOPSO/D) only replace individual solutions among subproblems; this paper extends genetic algorithm operators to information exchange between neighboring subproblems, performing crossover and mutation to improve population diversity.

## 2. Problem Formulation

### 2.1. Receiving Model and Analysis

In receiver array design, system receiving capability is determined by the far-field radiation pattern, which depends on element coordinates and excitation. The far-field pattern is the product of the element factor and the array factor [17]. It is a combination of individual element characteristics and element arrangement.

**Figure 1:**
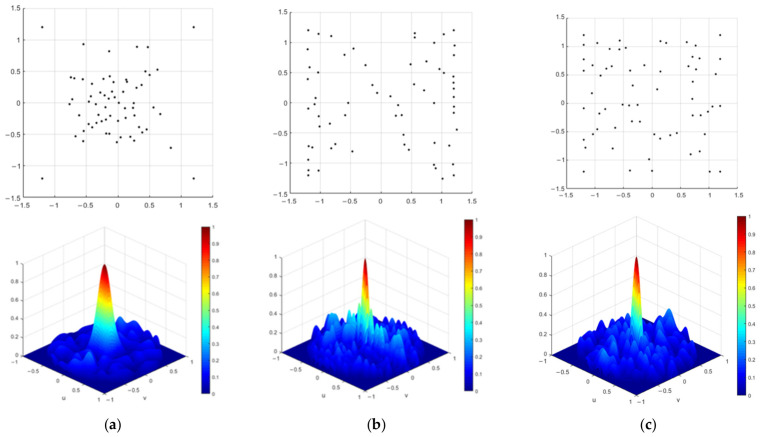
Radiation patterns of 64-element arrays with different layouts. (**a**) Array 1: MSL = −12.41 dB, G = 20.17 dB, BW = 11.82 deg; (**b**) Array 2: MSL = −6.10 dB, G = 20.96 dB, BW = 4.73 deg; (**c**) Array 3: MSL = −11.55 dB, G = 21.20 dB, BW = 6.21 deg.

In the Cartesian coordinate system, taking the positive x as the reference, the clockwise angle is defined as the azimuth angle φ, and the inclination angle relative to the xoy plane is defined as the elevation angle θ. To simplify array pattern calculation, the u−v coordinate system is introduced, where parameters u and v are mapped from azimuth and elevation angles via trigonometric transformation, and the far-field radiation pattern of the array at the specified scanning angle $\left(\theta_{0},\varphi_{0}\right)$ is derived as follows:

(1) $$\begin{array}{c} f\left(u,v\right)=\frac{1}{N}\sum_{n=1}^{N}E_{n}\left(u,v\right)I_{n}e^{jk\left(x_{n}\left(u-u_{0}\right)+y_{n}\left(v-v_{0}\right)\right)}\\ \left\{\begin{array}{c} u=sin\theta \cos\varphi \;u_{0}=\sin\theta_{0}\cos\varphi_{0}\\ v=\sin\theta \sin\varphi \;v_{0}=\sin\theta_{0}\sin\varphi_{0} \end{array}\right. \end{array}$$

where $\left(x_{n},y_{n}\right)$ and $I_{n}$ are, respectively, the two-dimensional planar coordinates and amplitude excitation of the n-th element; $k=\frac{2\pi }{\lambda }$ denotes the wave number; N stands for the total number of array elements. This factor normalizes the array summation result from the perspective of the number of array elements so that the response in the steering direction reflects the average contribution of each array element, thereby eliminating the linear influence of the number of array elements on the response amplitude. $E_{n}\left(\theta ,\varphi \right)$ denotes the element factor which characterizes the physical properties of a single array element; it can be derived from the transformation relations that substitute $\theta =\arcsin\left(\sqrt{u^{2}+v^{2}}\right)$ and $\varphi =a\tan2\left(v,u\right)$ into the element factor, yielding its representation $E_{n}\left(u,v\right)$ in the u−v coordinate system. The array factor reflects the influence of spatial geometric arrangement of multiple elements on array radiation performance. As shown in Figure 1, the core of array design is to adjust element positions and excitations to regulate key indicators, including MSL, G, and BW, so as to realize beamforming and meet diverse practical requirements.

The preference for conflicting indicators depends on receiving purposes and signal processing requirements, including strong interference suppression [18], high-precision direction finding with narrow BW [19], and balanced multi-target signal recognition.

**Figure 2:**
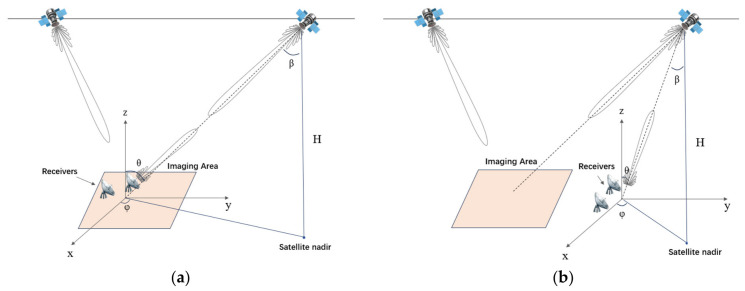
Schematic diagram of spaceborne SAR receiving modes (**a**) narrow-domain receiving mode; (**b**) wide-domain receiving mode.

Two receiving modes, narrow-domain and wide-domain receiving, shown in Figure 2, are classified by their relative spatial positions. The spaceborne SAR operates in a low Earth orbit and images a certain area on the ground. The satellite orbits at an altitude H, which is defined as the straight-line distance between the satellite and its subpoint on the Earth’s surface, with a slant range $R_{c}$ to the ground receiver. The transmit power and transmit antenna gain are denoted as $P_{t}$ and $G_{t}$, respectively. The receiving array is assumed to be ground-based. Typically, the north direction is taken as the positive y-axis, the east direction as the positive x-axis, and the vertical upward direction as the positive z-axis, forming a coordinate system. Affected by SAR transmit beam sidelobe suppression, wide-domain receiving obtains much lower received power, so higher receiving gain is required to satisfy the signal detection threshold [20]. Moreover, as the elevation angle θ of the satellite increases, the difficulty of signal reception becomes greater, which demands an even higher receiving gain.

According to the free-space propagation link budget of spaceborne SAR signals, the incident power density at the ground receiver can be expressed as:

(2) $$S=\frac{P_{t}G_{t}}{4\pi R_{c}^{2}L}$$

where L is the total link loss of the system. Based on the equivalent aperture principle of receiving antennas, the output power of the receiver array is given by:

(3) $$P_{r}=S\cdot A_{e}\cdot G\geq S_{i\min}$$

where $A_{e}$ denotes the effective aperture of a single array element, and G stands for the gain of the receiving array. To ensure successful signal detection, the received power $P_{r}$ must be greater than the minimum detectable signal $S_{i\min}$. The G requirement expressions for both modes are derived via antenna theory [21] and spatial geometric relations:

(4) $$\left\{\begin{array}{c} G\geq G_{rn}=\frac{{\left(4\pi \right)}^{2}LH^{2}}{S_{i\min}P_{t}G_{t}\lambda^{2}\cos^{2}\theta }\;\\ G\geq G_{rw}=\frac{{\left(4\pi \right)}^{2}LH^{2}}{S_{i\min}P_{t}G_{t}\lambda^{2}\cos^{2}\theta \eta_{0}} \end{array}\right.$$

where $\eta_{0}$ represents the attenuation correction coefficient of satellite signals under wide-area reception. In engineering practice, the amplified received signal must exceed the threshold to avoid being overwhelmed by internal noise. The required gain is determined by signal strength and scanning angle. Wide-angle receiving demands higher G, which raises the difficulty of array design and optimization.

### 2.2. Multi-Objective Optimization Model for Receiver Array

The multi-objective optimization of receiving arrays performs collaborative optimization on the two-dimensional coordinates and excitation amplitudes of N array elements within a fixed planar aperture of dimension $\left(L_{x},L_{y}\right)$ to optimize the multi-objective cost function of the synthesized radiation pattern under wide-angle scanning conditions. The decision vector is defined as:

(5) $$\begin{array}{cc} \left\{\begin{array}{c} X=\left[x_{1},y_{1},x_{2},y_{2},\ldots ,x_{N},y_{N}\right]\\ I=\left[I_{1},I_{2},\ldots ,I_{N}\right]\;\;\;\; \end{array}\right. & \begin{array}{c} \;\left(x_{n},y_{n}\right)\in [\frac{-L_{x}}{2},\frac{L_{x}}{2}]\times [\frac{-L_{y}}{2},\frac{L_{y}}{2}]\\ I_{n}\in [0,1] \end{array} \end{array}$$

The optimization problem of this paper can be defined as:

(6) $$\begin{array}{c} \underset{X,I\in \Omega }{Minimize}\left\{G_{agg}\left(X,I\left|\gamma \right.\right)=g\left(\gamma ,F\left(X,I\right)\right)\right\}\\ subject\;to\;e\left(X,I\right)\leq 0\; \end{array}$$

where $G_{agg}$ is the objective function, γ is the weight vector, and Ω is the feasible decision space. e includes position and excitation constraints, while F denotes the multi-objective vector, expressed as:

(7) $$\begin{array}{c} \underset{X,I\in \Omega }{Minimize}\left\{F\left(X,I\right)={\left[f_{1},f_{2},f_{3}\right]}^{T}\right\}\\ \left\{\begin{array}{c} f_{1}=MSL\left(X,I\right)\\ f_{2}=-G\left(X,I\right)\\ f_{3}=BW\left(X,I\right) \end{array}\right. \end{array}$$

To conclude, this work employs multi-objective optimization for element arrangement design, with the detailed approach introduced in Section 2. To satisfy engineering feasibility, a gradient-based soft penalty strategy is adopted for arrays whose inter-element spacing fails to meet the layout constraint $\sqrt{{\left(x_{i}-x_{j}\right)}^{2}+{\left(y_{i}-y_{j}\right)}^{2}}\geq d_{\min}$, where $d_{\min}$ generally takes a value within $\left[0,\;0.5\lambda \right]$. A penalty term will be appended to the objective function once the practical element spacing is smaller than $d_{\min}$.

(8) $$p=\min\left(\sum_{i=1}^{N-1}\sum_{j=i+1}^{N}c\cdot \max\left(0,\frac{d_{\min}-d_{ij}}{d_{\min}}\right),p_{\max}\right)$$

where c denotes the penalty coefficient and $p_{\max}$ represents the maximum limit of the penalty term. When the element spacing $d_{i}\geq d_{\min}$, the penalty term is zero, indicating no penalty; when $d_{i}<d_{\min}$, the penalty term becomes positive, and the more severe the violation, the larger the penalty. The individual penalties are summed, multiplied by the coefficient c, and then capped by the upper bound $p_{\max}$ to ensure dimensional consistency and controllable influence. This method flexibly adjusts the penalty strength, prevents the penalty term from dominating the optimization process and further suppressing population diversity, and realizes a balanced trade-off between performance indicators and physical layout for practical engineering.

### 2.3. Optimization Performance Indicators of Receiver Array

The core performance indicators in the design and optimization of the receiver array receiving system include MSL, G, and BW. After determining the positions and excitations of the array elements, the array pattern can be calculated, from which all these indicators can be extracted.

MSL is defined as the ratio of the maximum level in the sidelobe region to the mainlobe peak level. To define the sidelobe region S, the mainlobe exclusion region M must be specified first. Let $\left(u_{0},v_{0}\right)$ denote the direction of the mainlobe peak in the $\left(u,v\right)$ coordinate system. Along the u-direction, the two points where the normalized power pattern drops by 3 dB relative to the peak are found, denoted as $u_{\min}$ and $u_{\max}$. Similarly, along the v-direction, the 3 dB points are denoted as $v_{\min}$ and $v_{\max}$. The mainlobe region M is then defined as the rectangular area:

(9) $$M=\left\{\left.\left(u,v\right)\right|u_{\min}\leq u\leq u_{\max},v_{\min}\leq v\leq v_{\max}\right\}$$

The sidelobe region S is defined as the entire $\left(u,v\right)$ space, excluding the mainlobe region M.

The sidelobe level determines the anti-interference and clutter suppression capability of the receiver array system, which should be minimized in antenna synthesis design. Accordingly, MSL is expressed as:

(10) $$MSL=\underset{u,v\in S}{\max}\left|\frac{f\left(u,v\right)}{f_{\max}\left(u_{0},v_{0}\right)}\right|$$

G is a key indicator for evaluating the receiving capacity and directivity of arrays, which is generally calculated indirectly from the normalized radiation pattern under the assumption that the array elements are isotropic point sources [22]:

(11) $$G=D\times \eta =\frac{4\pi f^{2}\left(\theta_{0},\varphi_{0}\right)\eta }{\int_{0}^{2\pi }\int_{0}^{\pi }f^{2}\left(\theta ,\varphi \right)\sin\theta d\theta d\varphi }=\frac{4\pi \eta }{\int_{0}^{2\pi }\int_{0}^{\pi }f^{2}\left(\theta ,\varphi \right)\sin\theta d\theta d\varphi }$$

where D denotes the directivity coefficient of the receiver array, and η=0.7 represents the gain correction factor, which refers to the pattern radiation efficiency in antenna systems. The integration limits for θ and φ are $\left[0,\pi /2\right]$ and $\left[0,2\pi \right]$, respectively, corresponding to the upper-hemisphere radiation. Note that the performance indicators MSL and G are presented in decibels (dB) throughout this paper. For an MSL defined as a field amplitude ratio, its linear value is converted to dB via $MSL_{dB}=20\log_{10}\left(MSL_{linear}\right)$; for the G, which is a power ratio, the conversion is $G_{dB}=10\log_{10}\left(G_{linear}\right)$.

BW is traditionally defined as the angular range between the two −3 dB points on the mainlobe. In spaceborne SAR signal reception, the elevation angle of the satellite typically varies significantly as it flies over the receiving station, while the azimuthal change within the receiving beam is relatively minor. Therefore, this paper focuses on the BW in the θ direction as the key performance metric. Following the same parameter extraction concept as in the $\left(u,v\right)$ coordinate system, the peak direction $\left(\theta_{0},\varphi_{0}\right)$ is first located in spherical coordinates. By fixing the azimuth angle and scanning the elevation angle θ, the two angles $\theta_{\min}$ and $\theta_{\max}$ corresponding to the −3 dB points on either side of the normalized pattern peak are identified. The BW is then calculated as:

(12) $$BW=\theta_{\max}-\theta_{\min}$$

A narrower BW improves adjacent target resolution. Array design should comprehensively balance the three indicators and adjust priorities to achieve optimal receiving performance.

## 3. Design and Optimization of Receiver Array

### 3.1. Array Initialization

In array design and pattern synthesis, Taylor distribution combines ideal space factors with planar apertures. On the basis of linear array Taylor weighting, it is extended to two dimensions by normalized origin distance. Excitation varies regularly from the array center to edge and can be allocated to randomly positioned elements.

(13) $$I_{n}=1+2\sum_{m=1}^{\bar{n}-1}F_{m}\cos\left(m\pi \frac{r_{n}}{r_{\max}}\right)\;\;r_{n}=\frac{r_{i}}{r_{\max}}=\frac{\sqrt{x_{i}^{2}+y_{i}^{2}}}{r_{\max}}$$

where $F_{m}$ denotes the space factor:

(14) $$F_{m}=\frac{{\left(-1\right)}^{m+1}\prod_{k=1}^{\bar{n}-1}\left[1-\frac{m^{2}}{\sigma^{2}\left(k^{2}+{\left(A-0.5\right)}^{2}\right)}\right]}{\prod_{k=1,k\neq m}^{\bar{n}-1}\left(1-\frac{m^{2}}{k^{2}}\right)}\;\sigma =\frac{\bar{n}}{\sqrt{A^{2}+{\left(\bar{n}-0.5\right)}^{2}}}$$

where σ denotes the scaling factor, A is the Taylor parameter, and $\bar{n}$ is the number of equal-sidelobe nulls. Array initialization offers a favorable search starting point for subsequent optimization algorithms.

### 3.2. Receiver Array Multi-Objective Optimization Based on MOEA/D-HPSO

MOEA/D is a decomposition-based multi-objective evolutionary algorithm. It divides complex multi-objective problems into multiple single-objective subproblems by weight assignment and optimizes them concurrently [23]. This paper adopts HPSO for global search and introduces crossover and mutation of adjacent subproblems to enrich solution distribution, so that solutions can be evenly distributed on the Pareto front.

#### 3.2.1. MOEA/D Decomposition Framework

The decomposition of multi-objective optimization problems relies on appropriate aggregation functions for objective scalarization to construct single-objective subproblems [24]. The traditional weighted-sum function generates uniform weights via the Das–Dennis method according to the number of subproblems, which satisfies $\sum \gamma_{i}=1$. In 3D space, uniform sampling is performed on the plane $\gamma_{1}+\gamma_{2}+\gamma_{3}=1$, such that the weight vector $\left[\gamma_{1},\gamma_{2},\gamma_{3}\right]$ covers all directions in the objective space. The weighted-sum (WS) aggregation function is defined as:

(15) $$g^{ws}\left(X,I|\gamma \right)=\left[f_{1},f_{2},f_{3}\right]\cdot \left[\begin{array}{c} \gamma_{1}\\ \gamma_{2}\\ \gamma_{3} \end{array}\right]=\sum \gamma_{i}\cdot f_{i}$$

The Tchebycheff (TCH) aggregation function handles arbitrary Pareto front shapes [25]. It introduces a dynamic reference point z to shift the metric origin, mapping the function to any point on non-convex Pareto regions. The function is defined as:

(16) $$g^{TCH}\left(X,I|\gamma ,z\right)=\max\left\{\gamma_{i}\left|f_{i}-z_{i}\right|\right\}$$

For different weights on the Pareto front, each x is a Pareto optimal solution.

Aggregation functions scalarize multi-objective vectors. For a non-convex Pareto front, WS fails to cover concave non-dominated solutions, while TCH depends on reference points. Wide-angle SAR array optimization involves both a non-convex Pareto front and a highly multimodal fitness landscape, which cannot be effectively solved using a single aggregation strategy. Therefore, this paper combines both: WS rapidly explores the optimal region to build a reliable reference point, and then TCH guides local search for full Pareto coverage, improving solution distribution.

#### 3.2.2. Hybrid Genetic Particle Swarm Optimization (HPSO)-Based Array Solving Method

This paper adopts a hybrid search mechanism combining particle swarm optimization (PSO) and genetic algorithm (GA) operators to solve the decomposed single-objective subproblems. PSO is suitable for non-convex, non-smooth problems, with velocity and position updates defined as:

(17) $$\begin{array}{c} v_{i}\left(t+1\right)=\omega \left(t\right)\cdot v_{i}\left(t\right)+c_{1}r_{1}\left(pbest_{i}\left(t\right)-x_{i}\left(t\right)\right)+c_{2}r_{2}\left(gbest\left(t\right)-x_{i}\left(t\right)\right)\\ x_{i}\left(t+1\right)=x_{i}\left(t\right)+v_{i}\left(t+1\right) \end{array}$$

where $pbest_{i}\left(t\right)$ and $gbest\left(t\right)$ are the individual and global best positions, $c_{1}$ and $c_{2}$ are the cognitive and social learning factors, $r_{1},r_{2}\in \left[0,1\right]$ introduce randomness, and $\omega \left(t\right)$ is the inertia weight. To balance exploration and exploitation, a linearly decreasing inertia weight strategy is adopted:

(18) $$\omega \left(t\right)=\omega_{start}-\left(\omega_{start}-\omega_{end}\right)\cdot \frac{t}{T_{\max}}$$

The traditional MOEA/D adjacent information exchange promotes subproblem cooperation via fixed neighborhoods and direct replacement [26,27] but lacks genetic-level communication, which risks premature population convergence and diminishes subproblem specificity. Thus, the MOEA/D-HPSO framework hybridizes GA operators with the PSO-based main search to exchange information between adjacent subproblems. For the i-th subproblem, the neighborhood set is defined by the K-nearest subproblems in Euclidean distance of the weight vector. Each forms a parent pair $\left[p_{1},p_{2}\right]$, and the SBX operator performs simulated binary crossover on them with a certain probability. The d-th dimension of the generated offspring is given by:

(19) $$\begin{array}{l} c_{1,d}=0.5\left[\left(1+\beta \right)p_{1,d}+\left(1-\beta \right)p_{2,d}\right]\\ c_{2,d}=0.5\left[\left(1-\beta \right)p_{1,d}+\left(1+\beta \right)p_{2,d}\right] \end{array}\;\;\;\beta =\left\{\begin{array}{l} \begin{array}{cc} {\left(2u\right)}^{1/\left(\eta_{c}+1\right)} & \;\;\;\;\;u\leq 0.5 \end{array}\\ \begin{array}{cc} {\left(\frac{1}{2\left(1-u\right)}\right)}^{1/\left(\eta_{c}+1\right)} & u>0.5 \end{array} \end{array}\right.$$

where $u∼U\left(0,1\right)$. The offspring then undergoes polynomial mutation with a given probability.

(20) $${c^{\prime }}_{1,d}=c_{1,d}+\delta \cdot \left(u_{b}-l_{b}\right)\;\;\;\delta =\left\{\begin{array}{cc} {\left(2u\right)}^{1/\left(\eta_{m}+1\right)}-1 & u\leq 0.5\\ 1-{\left(2\left(1-u\right)\right)}^{1/\left(\eta_{m}+1\right)} & u>0.5 \end{array}\right.$$

This introduces GA fine-tuning at the genetic level in the PSO-dominated framework, maintaining stable search direction while enhancing population diversity and local exploitation.

### 3.3. Algorithm Implementation Details

To ensure the reproducibility of the proposed MOEA/D-HPSO array solver, this section provides a detailed description of its algorithmic components and complete pseudo-code, as summarized in Algorithm 1.

**Algorithm 1** MOEA/D-HPSO for planar receiving array optimization**Input:** number of elements $N=N_{x}\times N_{y}$, aperture size $L_{x}\times L_{y}$, number of subproblems $N_{p}$, neighborhood size *K*, maximum generations $T_{\max}$, population size per subproblem *M*.**Output:** Pareto front P, HV history $hv_{history}$
1: weights ← Generate Uniform Weights ($N_{p}$, 3);  // 3 objectives decision vector: $X=\left[x_{1},y_{1},x_{2},y_{2},\ldots ,x_{N},y_{N}\right],\;I=\left[I_{1},I_{2},\ldots ,I_{N}\right]$ with $x_{n}\in \left[-L_{x}/2,L_{x}/2\right],\;y_{n}\in \left[-L_{y}/2,L_{y}/2\right],\;I_{n}\in \left[0,1\right]$2: Compute distance matrix among weights;3: **for** each subproblem *j*, set neighbor list $B\left(j\right)$ as the *K* closest weight vectors;4: $z=\left(+\infty ,+\infty ,+\infty \right)$;  // initialize ideal point5: $t_{start}\leftarrow floor(T_{\max}/2)$;  // generation to switch to Tchebycheff aggregation6: **for** each subproblem *j*
**do**:7:  ${pop}_{j}\leftarrow$ InitializePopulation (*M*);  // each individual generated with Taylor + noise8:  Define fitness function $F_{j}$ using current aggregation: weighted sum (if gen < $t_{start}$) else Tchebycheff (if gen ≥ $t_{start}$), plus position penalty;9:  Initialize a PSO instance for subproblem *j* with ${pop}_{j}$ and $F_{j}$;10:  Store best solution ${pbest}_{ij}$ and its objectives;11: **end for**12: Initialize an empty list ${hv}_{history}$;  //store the HV history obtained in each generation13: **for** gen = 0 to $T_{\max}-1$
**do**:14:  **for** each subproblem *j* in parallel **do**:15:   Perform one PSO generation: update velocity, position, personal best ${pbest}_{ij}$ and global best ${pbest}_{j}$ (best solution among all subproblems in $B\left(j\right)\cup \left\{j\right\}$);16:  **end for**17:  **if** gen % 10 == 0 and gen > 0:18:   **for** each subproblem *k* do:19:    *k* ← random neighbor from $B\left(j\right)$;20:    Apply SBX crossover and polynomial mutation to $p_{j}$ and $p_{k}$;21:    Replace the worst individual in subproblem *j*’s swarm with the better child (evaluated by $F_{j}$);22:   **end for**23:  **end if**24:  Update ideal point *z* using best objectives of all subproblems;25:  **if** gen % 5 == 0 or gen == $T_{\max}-1$:26:   Compute hypervolume HV from current non-dominated solutions;27:   Append HV to ${hv}_{history}$;28:  **end if**29: **end for**30: Collect all subproblem best solutions and their raw objectives;31: Perform non-dominated sorting on [MSL, -G, BW] to extract Pareto front P;32: Return P and ${hv}_{history}$;

Each individual encodes 3N−8 variables. The four corner elements are fixed to maintain the aperture size. During initialization, internal positions are randomly generated, and excitation amplitudes are obtained from a Taylor distribution plus Gaussian noise. Each subproblem maintains a PSO swarm. Each particle stores its personal best. The global best used in velocity update is taken as the best individual among the subproblem itself and its K neighbors. Each velocity component is clipped to the interval $\left[-v_{\max},v_{\max}\right]$ (position: [−0.15,0.15], excitation: [−0.049,0.049]). Every 10 generations, simulated binary crossover and polynomial mutation (mutation probability $p_{m}=0.1$, distribution index $\eta_{m}=20$, crossover probability $p_{c}=0.9$, distribution index $\eta_{c}=20$) are applied to the two global best individuals, and the better offspring replaces the worst individual in the swarm. The ideal point z is updated each generation as the component-wise minimum. An external archive is maintained via non-dominated sorting. Hypervolume is computed every five generations from the archive after normalization. The algorithm runs for $T_{\max}=300$ generations. The final Pareto front is extracted by non-dominated sorting on [MSL, -G, BW].

## 4. Experimental Results and Analysis

In this work, the wavelength of the intercepted signal is set to λ=0.3m. The planar array uses fixed corner elements to maintain the aperture size $L_{x}\times L_{y}=10\lambda \times 10\lambda$ under a scanning range of $\pm 60^{\circ }$. Arrays with N=100 and N=64 elements are designed. The MOEA/D parameters are set as follows: 100 subproblems, internal population size of 20, maximum iterations of 300, and information exchange every 10 iterations. The final Pareto front is obtained after multiple independent runs.

### 4.1. Pareto Front Analysis and Comparison

To verify the effectiveness of the proposed MOEA/D-HPSO for the on-board SAR receiver array optimization, it is compared with traditional methods, including NSGA-II, MOPSO/D [28], MOEA/D-DE, and MO-GPSOD [29]. NSGA-II employs a non-decomposition framework based on crowding distance and does not employ the decomposition-based subproblem partition strategy or the hybrid PSO search mechanism. MOPSO/D adopts a single-population particle swarm optimization under the decomposition framework and does not set up independent swarms for each subproblem. MOEA/D-DE uses differential evolution as the subproblem solver, which is distinctly different from the hybrid particle swarm optimization that incorporates a genetic neighbor exchange strategy. MO-GPSOD combines geometric particle swarm optimization with decomposition but does not adopt the proposed hybrid aggregation method and genetic operators. All methods in this paper were executed under the same hardware conditions and parameter settings. Random seeds are generated according to system time to guarantee independent runs for every experiment. NSGA-II uses a population size of 100. For MOEA/D-DE, the differential evolution parameters are set to F=0.5 and CR=1. All other parameters for the three comparison algorithms (e.g., number of generations, neighborhood size, mutation probability, and for MOPSO/D, the inertia weight, learning factors, and velocity clamping) are identical to those of the proposed MOEA/D-HPSO described in Section 3. Each algorithm runs independently three times, with a maximum of 300 iterations per run. Upon completion of all runs, the solutions obtained from the three runs are integrated into a single set. Non-dominated sorting is then performed on this integrated set, and the non-dominated individuals are retained to form the final Pareto front. Here, a non-dominated solution refers to an individual for which no other solution is no worse than it across all objectives and superior to it on at least one objective.

**Figure 3:**
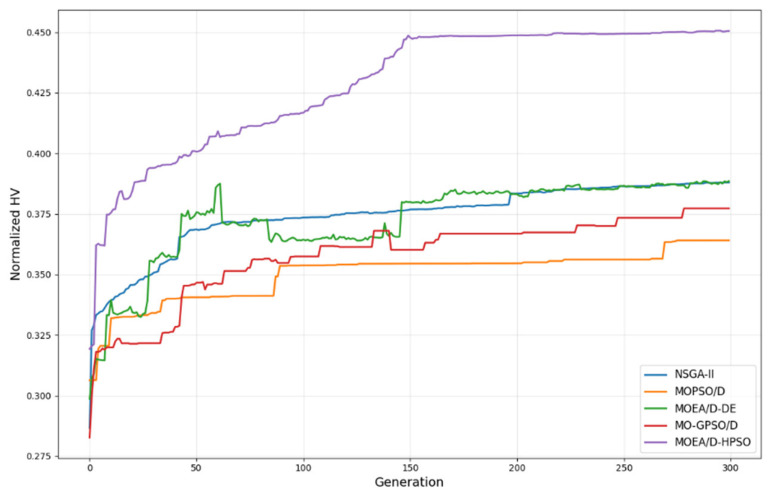
Normalized HV convergence curves.

Hypervolume (HV) is adopted as the primary metric to simultaneously evaluate the convergence and diversity of the obtained Pareto fronts. Given an approximate Pareto solution set and a reference point $r=\left(r_{1},r_{2},\ldots ,r_{m}\right)$ in the m-dimensional objective space, HV measures the volume of the union of hypercubes dominated by each solution with respect to r. In this paper, the three objective functions are $f_{1}=MSL$, $f_{2}=-G$, and $f_{3}=BW$. Thus, a lower value in each transformed objective is better. Each objective value $f_{i}$ is normalized to [0,1] using ideal and nadir points as normalization bounds:

(21) $${\hat{f}}_{i}=\frac{f_{i}-z_{i}^{ideal}}{z_{i}^{nadir}-z_{i}^{ideal}}$$

where the ideal vector is $z^{ideal}=\left[-20,-25,5\right]$ and the nadir vector is $z^{nadir}=\left[0,15,30\right]$. To ensure the comparability of results from different algorithms and the stability of evaluation, fixed bounds rather than dynamic ones are adopted in this paper. The original objective functions are normalized with the $z^{ideal}$ and $z^{nadir}$, and all normalized objective values are clipped to the interval $\left[0,1\right]$. Afterwards, taking $r=\left[1,1,1\right]$ as the reference point in the normalized objective space, the HV indicator is calculated via the PyMOO toolkit. Only the non-dominated solutions of each approximated Pareto front are considered. A higher HV value indicates a better overall approximation to the true Pareto front. Figure 3 shows the normalized HV trends over 300 iterations.

To ensure the comparability of results from different algorithms and the stability of evaluation, fixed reference points rather than dynamic ones are adopted in this paper. The original objective functions are normalized with the $z^{ideal}$ and $z^{nadir}$, and all normalized objective values are clipped to the interval $\left[0,1\right]$. Afterwards, taking $r=\left[1,1,1\right]$ as the reference baseline in the normalized objective space, the hypervolume indicator is calculated via the PyMOO toolkit (version 0.6.1.5).

As shown in Figure 3, after optimization, the final hypervolume (HV) value achieved by the proposed MOEA/D-HPSO algorithm is 0.4505, while those of NSGA-II, MOPSO/D, MOEA/D-DE, and MO-GPSO/D are 0.3879, 0.3641, 0.3885, and 0.3773, respectively. Compared with the four benchmark algorithms, the HV of the proposed algorithm is improved by 16.1%, 23.7%, 15.9%, and 19.4%, respectively, demonstrating its significant advantages in convergence accuracy and solution set diversity. From the convergence curve, the HV of the proposed algorithm rapidly increases to approximately 0.42 within the first 50 generations, exhibiting strong global exploration capability; after about 150 generations, the curve stabilizes, and the final HV reaches 0.4505, reflecting a good balance between convergence and diversity. In contrast, the HV of MOPSO/D consistently fluctuates between 0.36 and 0.37, indicating slow convergence and severe lack of diversity; the HV curves of MOEA/D-DE and NSGA-II exhibit significant fluctuations around 0.38–0.39, suggesting uneven solution set distribution; although MO-GPSO/D converges quickly in the early stage, its final HV is only 0.3773, implying poor diversity. Furthermore, examining the last 50 generations, the proposed algorithm achieves a mean HV of 0.4502 with a standard deviation of only 0.00015, indicating excellent stability near the optimum. In comparison, NSGA-II yields a mean of 0.3874 ± 0.00054, MOPSO/D gives 0.3639 ± 0.0021, MOEA/D-DE yields 0.3880 ± 0.00068, and MO-GPSO/D gives 0.3772 ± 0.00012 (mean ± std). The low variance of the proposed method confirms its robust convergence, whereas the higher variance of MOPSO/D and MOEA/D-DE reflects ongoing oscillations and difficulty in settling to a stable Pareto front. In summary, the proposed algorithm outperforms the other four mainstream multi-objective optimization algorithms in terms of convergence speed, final solution set quality, and stability.

**Figure 4:**
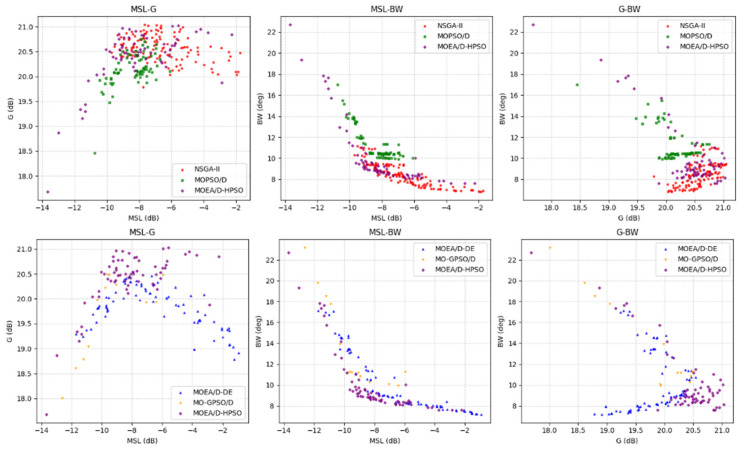
Two-dimensional Pareto front distributions of five algorithms; scanning angle: $\pm 60^{\circ }$, N=100.

Figure 4 compares the Pareto solution distributions of the five algorithms. MOPSO/D and NSGA-II solutions are concentrated within a narrow 4 dB MSL range (−10 dB to −6 dB), failing to form a complete Pareto front. MO-GPSO/D achieves an MSL range of 11 dB and an inter-quartile spread of 3.2 dB, indicating good diversity, but it acquires only few non-dominated solutions, insufficient to support diverse engineering decisions, revealing its deficiency in diversity preservation. The best MSL, G, and BW of MO-GPSO/D are −10.71 dB, 20.4 dB, and 7.2°, respectively.

Compared to the proposed method’s best values (−13.7 dB, 21 dB, 7.6°), MO-GPSO/D is 2.99 dB worse in MSL, 0.6 dB worse in G, and only 0.4° better in BW, presenting inferior overall optimization. In summary, the proposed algorithm’s Pareto solutions span a 12 dB MSL range (from −13.7 to −1.7 dB)—three times wider than that of NSGA-II/MOPSO/D—and are located significantly closer to the region of low MSL, high G, and narrow BW. It provides substantially more non-dominated solutions than MO-GPSO/D, offering more feasible trade-offs under conflicting multi-objective constraints, which verifies its superiority in complex optimization problems.

In other words, a concentrated front locks the receiver into a fixed performance trade-off, failing to support conflicting requirements such as anti-jamming versus high sensitivity. A widely distributed front, by contrast, provides explicit scenario-tailored options, making the proposed method inherently more adaptable to diverse spaceborne SAR receiving conditions.

### 4.2. Array Design Results Under Different Receiving Conditions

**Figure 5:**
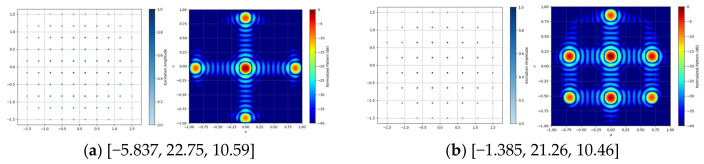
Element scatter diagrams, radiation patterns, and corresponding performance indicators [MSL (dB), G (dB), BW (deg)] for uniformly filled rectangular arrays with Taylor-weighted excitation: (**a**) N=100; (**b**) N=64.

To illustrate the grating lobe issue of uniform arrays under wide-angle scanning, Figure 5 first presents the design results of uniform arrays with Taylor amplitude weighting.

Under wide-angle scanning, severe grating lobes appear in multiple directions, which prevents the receiving system from effectively distinguishing real signals from interference in the grating lobe directions, leading to angular ambiguity and reception failure. Specifically, the MSL of the 100-element uniform array is −5.837 dB, while that of the 64-element uniform array is as high as −1.835 dB, indicating that the grating lobes are almost as strong as the mainlobe, severely degrading beam quality. Furthermore, uniform arrays lack flexibility in beam shape control and cannot simultaneously suppress grating lobes while achieving multiple performance indicators such as high G and narrow BW, thus failing to meet the diverse receiving requirements under wide-angle scanning for spaceborne SAR.

**Figure 6:**
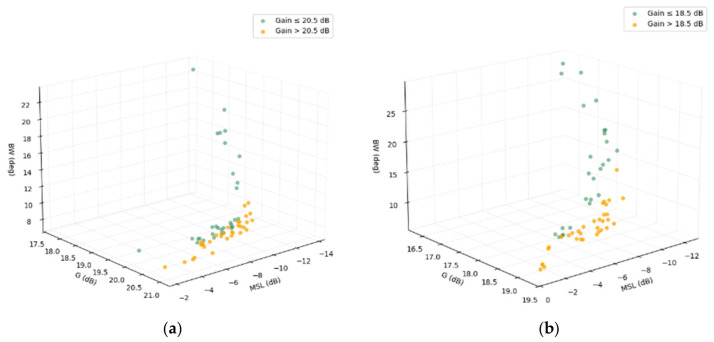
3D Pareto solution distributions. (**a**) N=100; (**b**) N=64.

Figure 6 presents the Pareto solutions obtained by MOEA/D-HPSO after 300 iterations under the scanning angle of $\pm 60^{\circ }$, with array elements of 100 and 64. The 100-element array achieves an MSL range of [−13.68, −2.25] dB, a G range of [17.68, 21.03] dB, and a BW range of [7.61, 22.69] deg, while the 64-element array yields [−12.37, −0.71] dB, [16.31, 19.29] dB, and [6.91, 28.18] deg, respectively. Thus, the 100-element array possesses higher design degrees of freedom, leading to a 1.31 dB improvement in best-case MSL and a 1.74 dB improvement in best-case G, with comparable best-case BW. This moderate concentration of the solution set still supports diverse engineering trade-offs. In wide-range receiving mode, the G constraints are set to 20.5 dB and 18.5 dB for the two arrays, respectively, and qualified solutions satisfying these constraints are screened out. Taking the two arrays as examples, several typical design schemes selected from the Pareto front are discussed according to different optimization priorities.

**Figure 7:**
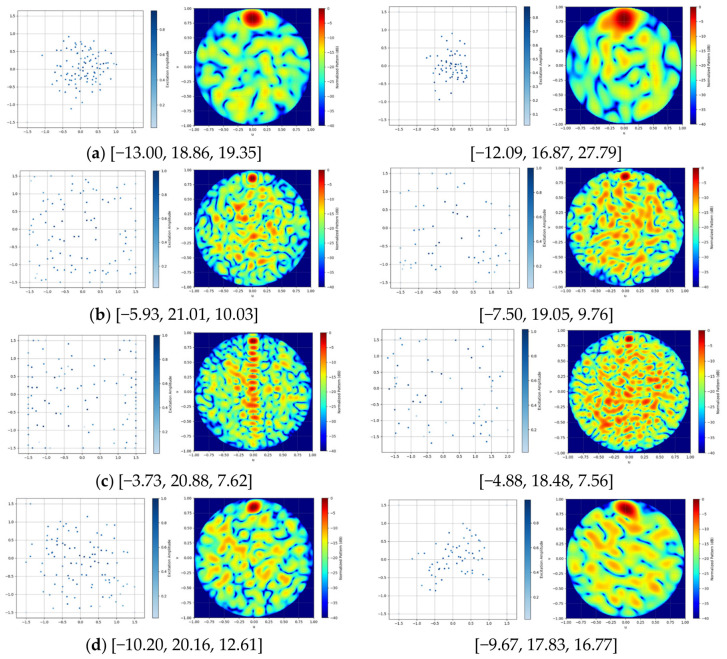

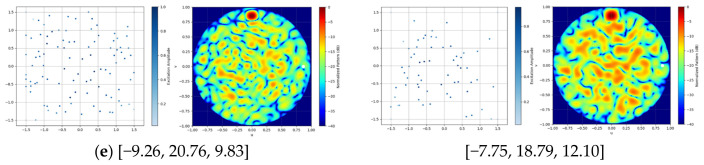
Typical array designs: (**a**) low-sidelobe design; (**b**) high-gain design; (**c**) narrow-beam design; (**d**) balanced multi-objective design; (**e**) wide-range receiving design. Each subfigure shows the element scatter diagram, radiation pattern, and corresponding performance indicators [MSL (dB), G (dB), BW (deg)].

It can be observed from Figure 7 that all displayed array designs with different optimization priorities are non-dominated solutions from the Pareto front. Scheme (a) prioritizes low MSL. The array elements are densely arranged to suppress grating lobes effectively under wide-angle scanning. Its radiation pattern features negligible energy leakage in undesired directions, making it suitable for signal receiving in strong interference scenarios with wide-angle scanning. Scheme (b) focuses on high G, which is applicable to receiving scenes with severe energy shortage. Restricted by objective conflicts, this design sacrifices sidelobe performance and BW. Scheme (c) is optimized for a narrow mainlobe with the highest angular resolution. According to the Rayleigh criterion, for ideal point targets whose angular extent is negligible compared to the BW, two targets can be distinguished when their angular separation satisfies $\left|\theta_{1}-\theta_{2}\right|\geq BW$. In this context, the beamwidth directly quantifies the minimum resolvable separation between nearby targets, and targets with angular extent much smaller than BW are effectively point-like. Similar to uniform arrays, it narrows the mainlobe at the cost of higher MSL. Scheme (d) realizes balanced multi-objective optimization for narrow-range receiving, achieving comprehensive performance balance among interference suppression, energy utilization, and angular resolution. Scheme (e) is selected from qualified solutions satisfying G constraints of 20.5 dB and 18.5 dB for wide-range receiving. It satisfies G requirements while coordinating other indicators and achieves favorable receiving performance.

## 5. Conclusions

This paper investigates the design of spaceborne SAR signal-receiving arrays under wide-angle scanning, establishes a multi-objective optimization model, and proposes a non-uniform planar array optimization method based on MOEA/D-HPSO.

The algorithm integrates decomposition and hybrid particle swarm optimization, uses hybrid aggregation functions to adapt to non-convex characteristics, and introduces genetic operations to enhance population diversity. It achieves better convergence and higher-quality Pareto solutions than conventional algorithms.

The designed non-uniform planar arrays suppress grating lobes and reduce MSL under wide-angle scanning while flexibly achieving high G and narrow BW. The proposed method handles conflicting indicators well, and the obtained Pareto solutions meet diverse receiving requirements in engineering.

## Data Availability

No new data were created or analyzed in this study.
